# Seasonal Dynamics of *Anaplasma* *phagocytophila* in a Rodent-Tick (*Ixodes trianguliceps*) System, United Kingdom

**DOI:** 10.3201/eid0901.020169

**Published:** 2003-01

**Authors:** Kevin J. Bown, Michael Begon, Malcolm Bennett, Zerai Woldehiwet, Nicholas H. Ogden

**Affiliations:** * University of Liverpool, Liverpool, United Kingdom

**Keywords:** *Anaplasma*, *Ehrlichia phagocytophila*, *Ixodes trianguliceps*, rodent, Europe, research

## Abstract

We investigated the reservoir role of European wild rodents for *Anaplasma phagocytophila* using polymerase chain reaction (PCR) analysis of blood collected from individually tagged rodents captured monthly over 2 years. The only tick species observed in the woodland study site was *Ixodes trianguliceps*, and ruminant reservoir hosts were not known to occur. *A. phagocytophila* infections were detected in both bank voles and wood mice but were restricted to periods of peak nymphal and adult tick activity. Most PCR-positive rodents were positive only once, suggesting that rodent infections are generally short-lived and that ticks rather than rodents may maintain the infection over winter. Bank voles were more likely to be PCR positive than wood mice, possibly because detectable infections are longer lived in bank voles. This study confirms that woodland rodents can maintain *A. phagocytophila* in Great Britain in the absence of other reservoir hosts and suggests that *I. trianguliceps* is a competent vector.

*Anaplasma phagocytophila* (formerly *Ehrlichia phagocytophila, E. equi,* and the agent of human granulocytic ehrlichiosis [HGE]; [*1*]) is an obligate intracellular bacterium that targets mainly granulocytes in its mammalian hosts (*2*). This bacterium has a wide mammalian host range, infecting domesticated animals such as dogs, sheep, cows, and horses (*2*–*5*), as well as wildlife species such as deer and rodents (*6**,**7*). The discovery of HGE, an acute febrile disease, in the United States and Europe (*8*,*9*) has generated increasing public health interest in this organism.

*A. phagocytophila* is transmitted by ixodid ticks; in the United States, the principal vectors are *Ixodes scapularis* and *I. pacificus* (*6*,*10*), while in Europe the main vector is thought to be *I. ricinus* (*3*). *A. phagocytophila* is transstadially transmitted by all these vector ticks and, to date, no evidence of transovarial transmission has been found (*3*,*6*,*11*,*12*).

A number of studies have reported *A. phagocytophila* infection in wild rodents in the United States, the United Kingdom, and mainland Europe (*6*,*13*,*14*), but relatively little is known about the precise role that rodents play in its ecology and epidemiology, especially in Europe. Recently, *A. phagocytophila* has been detected in woodland rodents in northwest England, where *I. trianguliceps*, a nidicolous tick that feeds almost exclusively on small mammals, was the only tick species identified on rodents (*11*). This woodland rodent/*I. trianguliceps*/*A. phagocytophila* system is therefore one of many supporting a tickborne zoonosis, where lack of knowledge of the dynamics of the interacting populations is a major barrier to understanding potential threats to human health. We report the results of a longitudinal study of this system conducted during 2 years from January 1997 to December 1998.

## Materials and Methods

### Small Mammals and Sample Collection

The study was conducted in woodland area in northwest England (N53:20:48, W03:02:50). Grazing livestock were excluded by fencing, and no deer are present in the locality. Brown hares (*Lepus europaeus*) and grey squirrels (*Sciurus vulgaris*) occur in the wood at low densities. Although their status as reservoir hosts for *A. phagocytophila* is unknown, these two species are unlikely to be frequent hosts of the nidicolous *I. trianguliceps*. Rodents were trapped as previously described (*15*,*16*). Briefly, 200 Longworth traps (Penlon Ltd, Oxfordshire, UK) were placed on a 1-hectare grid over 3 consecutive trap-nights every 4 weeks in 1997 and 1998 for a total of 26 sample periods. Individual animals were identified by a microchip transponder (Avid Pettrac, Sussex, UK), inserted subcutaneously on first capture. At first capture in each sample period, we recorded body mass, numbers of feeding ticks, and evidence of flea infestation and took a blood sample (approximately 50 µL) from the tail tip, after which the rodent was released. On first capture, we also assigned animals to one of three age categories on the basis of mass. Using monthly growth rates estimated from field data and laboratory information on mass at 2 weeks of age, we calculated mass thresholds for juvenile (J; <6weeks), subadult (S; 6–10 weeks) and adult (A; >10 weeks) age categories (unpub. data). Thresholds used were as follows: wood mice, April–July: J = <15 g, S = 15–18 g, A = >18 g; wood mice, August–December: J = <14 g, S = 14–17 g, A = >17 g; bank voles, April–July: J = <14 g, S = 14–17 g, A = >17 g; and bank voles, August–December: J = <12 g, S = 12–14 g, A = >14 g.

This study was intended to be as noninvasive as possible, and ticks were not routinely collected in case this affected the transmission of *A. phagocytophila* in the study site where pilot studies had suggested that tick densities were low. Small numbers of engorged ticks were, however, collected from rodents at the study site between May 1997 and August 1998. Because of the nidicolous nature of *I. trianguliceps*, no questing ticks were collected.

### DNA Extraction and Polymerase Chain Reaction (PCR)

DNA was extracted from blood pellets by alkaline digestion (*17*). We added 0.5 mL 1.25% ammonia solution to the blood sample in a Sure-Lock microcentrifuge tube (Fisher Scientific, Loughborough, UK) and heated at 100°C for 20 min. After brief centrifugation, the tubes were opened and heated until half the initial volume had evaporated. This solution was then diluted 1 in 10 in sterile deionized, distilled water, and 5 µL was included in the first round of PCR reactions. Sensitivity was compared with that of a commercial kit by extracting DNA from serial dilutions of acutely infected sheep blood; PCR of these dilutions indicated the limit of detection to be approximately two infected leukocytes for both methods, the same as previously reported (*11*). The same method was used to extract DNA from ticks that had first been macerated in the microcentrifuge tube with a pipette tip. For engorged adult female ticks, however, the initial volume of 1.25% ammonia solution was 1 mL.

*A. phagocytophila* infection was detected by using a nested PCR specifically targeting the 16S rDNA of *A. phagocytophila*, as described previously (*11*). Each 50-µL reaction contained 1.5 mM Mg Cl_2_, 0.2 mM each of dNTP, 75 mM Tris-HCl (pH8.8), 20 mM (NH_4_)_2_SO_4_, 1.25 U *Taq* polymerase (Abgene, Surrey, UK), and 40 pmol of each of the following primers (*18*):

first reaction: EE1: TCCTGGCTCAGAACGAACGCTGGCGGC; EE2: GTCACTGACCCAACCTTAAATGGCTG; second reaction: EE3: GTCGAACGGATTATTCTTTATAGCTTGC; EE4: CCCTTCCGTTAAGAAGGATCTAATCTCC. For the second-round reaction, 1 µL of the first-round product was added as template. Both reactions consisted of 35 cycles of 95°C for 30 sec, 55°C for 30 sec, and 72°C for 60 sec, followed by a final extension stage of 72°C for 5 min.

### 16S rRNA Sequence Analysis

The PCR product from a positive bank vole was cloned by using the TOPO TA cloning kit (Invitrogen Corp., Carlsbad, CA) and sequenced by using an ABI 377 automated sequencer. The sequence (GenBank accession no. AY082656) was compared to previously published *A. phagocytophila* sequences on GenBank by using the BLAST program from the National Center for Biotechnology Information website (available from: URL: http://www.ncbi.nlm.nih.gov/BLAST/).

### Statistical Analysis

We investigated two outcome variables, the numbers of ticks counted per rodent and the rodent blood PCR result. Our purpose was to identify the factors that influenced the contact rates of rodents with ticks and the probability that the rodents acquired *A. phagocytophila* infections.

### Analysis of Rodent Infestations

Distributions of larval, nymphal, and adult ticks were significantly different from normal and Poisson distributions (p<0.05), but none were significantly different from the negative binomial distribution (p>0.1). Consequently, factors influencing the numbers of adult, nymphal, and larval ticks counted per rodent were investigated by using negative binomial, linear regression models in STATA for Windows version 6 (*19*). Rodent ID number was included as a random effect to account for repeated sampling of some of the rodents (*20*).

The analysis was undertaken in two stages. In the first stage, we investigated any seasonal variations in the abundance of ticks because such variations could superimpose on seasonal variations in rodent demography and confound investigation of animal-level variables. This stage itself involved three steps. First, we tested the null hypothesis that no significant variation existed in the counted numbers of ticks among sample periods. Second, we tested the null hypothesis that variation in the numbers of ticks among sample periods was not different from the specific pattern of seasonal *I. trianguliceps* abundance suggested by the detailed study of Randolph (*21*) after visual examination of data from a woodland in southern England (N51:01:46; W0:50:11). Other studies on *I. trianguliuceps* conducted in different locations in the United Kingdom at different times have suggested that the seasonal pattern of *I. trianguliceps* abundance observed by Randolph is more general in the United Kingdom (*22*–*24*). In this analysis we compared the power of binary variables for year, season (spring, summer, autumn, and winter), and the tick activity periods observed by Randolph (e.g., June and September–January for larvae, and May–August for nymphs) to explain any between-sampling variation in tick abundance observed in the present study. Third, we sought the optimum pattern of between-sampling variation in tick abundance in the present study by investigating binary dummy variables for each sampling period, as well as those for year and seasons. In these models, forward and backward elimination and combination of variables were performed stepwise until a minimal model was reached beyond which the variables could not be combined without significantly affecting model deviance.

In the second stage, we investigated rodent-level factors of species, sex, age category, and mass as explanatory variables for tick infestations in multivariable models that accounted for any seasonal variation in tick abundance deduced in the first stage. Mass and age category were investigated in separate models because of some colinearity. We also investigated interactions between sex and species and between sex and mass as explanatory factors. In addition, evidence for relationships between parasitism with one tick developmental stage and another was investigated by using similar regression models, accounting for any deduced seasonal variation in tick abundance and notable animal-level factors. The critical probability was p<0.05 throughout.

### Analysis of PCR Result

Rodent species and sex, mass, and age category (in separate models), the presence of fleas (a binary variable), and the numbers of larval, nymphal, and adult ticks counted per rodent were investigated as variables that could explain results of PCR analysis of rodent blood by using logistic regression models in STATA. Interactions between sex and species and between sex and mass were also investigated as explanatory factors. The binary variable age (rodent >6 months old) was investigated in case susceptibility increased in older animals that had not recently received infectious challenge, as occurs in sheep (*2*). Rodent ID number was again included as a random effect. The likelihood that a rodent encountered a tick of a particular developmental stage in any month of the study was investigated by using variables developed (as described previously) in investigations of the seasonal activity of ticks. However, any infections detected at one sample period may have been acquired from ticks (or other vectors) that attached to rodents in the previous sample period (because of latent detectability of infections) (*3*,*25*–*27*), or from ticks that fed and dropped off without being counted (*21*). To account for this, we investigated four additional binary variables as explanatory of rodent infections: whether the rodent was infested, either at the time of sample or the previous sample period, with a tick of a given stage or a flea, termed “carried a larva,” “carried a nymph,” “carried an adult,” and “carried a flea.” The critical probability was p<0.05 throughout.

## Results

### Rodents Captured and Their Tick Infestations

Over the study period, we captured 690 rodents: 475 wood mice (*Apodemus sylvaticus*) and 209 bank voles (*Clethrionomys glareolus*), plus 6 field voles (*Microtus agrestis*) which, because of the low numbers, were excluded from subsequent analyses. *I. trianguliceps* was the only species of tick found on the rodents during this study. Data on the numbers of captures and ticks are summarized in Table 1.

**Table 1:** Summary data of the rodents captured and the numbers of attached ticks

	Bank voles (mean per rodent)	Wood mice (mean per rodent)	Totals (ratio, vole:mouse)
No. captures	597	1,368	1,965 (1:2)
No. larvae (mean per rodent)	125 (0.21)	368 (0.27)	493 (0.25) (1:3)
No. nymphs (mean per rodent)	57 (0.10)	30 (0.02)	87 (0.04) (2:1)
No. adult ticks (mean per rodent)	19 (0.03)^a^	18 (0.01)	37 (0.02) (1:1)

^a^One bank vole carried 10 adult female ticks.

We found significant differences among sampling periods in the numbers of larvae and nymphs counted on rodents (likelihood ratio statistic chi square=72 and 65 for larvae and nymphs, respectively, df=25, p<0.0005). We found no significant differences among sampling periods in the numbers of adults counted on rodents (chi square=22, df=25, p>0.25).

**Figure 1:**
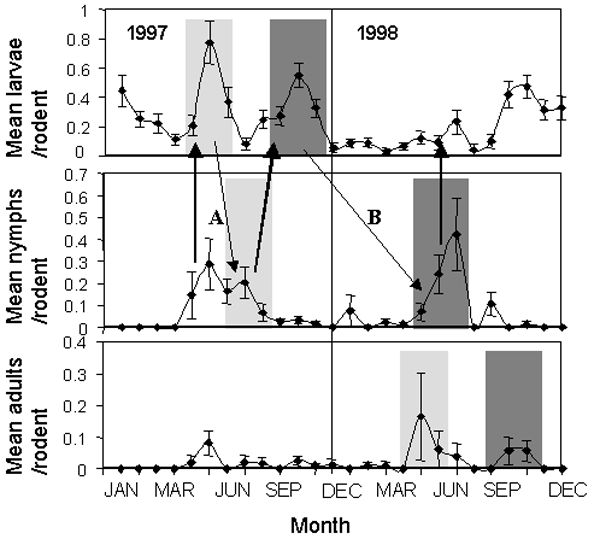
The mean (+/- SE) numbers of larval, nymphal, and adult *Ixodes trianguliceps* ticks counted per rodent at 4-week intervals, 1997–1998. Shaded areas of similar intensity indicate ticks of different instars that may have belonged to the same cohort, according to interstadial development times deduced by Randolph (*21*). Arrows indicate potential transmission cycles: bold arrows indicate potential transmission from infected nymphs to uninfected larvae by means of rodent infections. Fine arrows indicate potential transstadial transmission from infected engorged larvae to infected host-seeking nymphs. For clarity only one within-year (A) and one between-year (B) cycle involving nymphal and larval ticks are illustrated.

The counted numbers of larvae were significantly higher in those sample periods that occurred in months when larvae were most abundant in the studies of Randolph (coefficient=0.311, SE=0.140, p=0.027) (*21*). This, however, only partly explained the between-sampling variation in larval abundance: in the most parsimonious model, significantly more larvae were counted on the rodents in 1997 than in 1998 (coefficient=0.506, SE=0.114, p<0.001) and significantly more were counted in autumn than in other months (coefficient=0.568, SE=0.135, p<0.001). In the more detailed analysis, the most parsimonious model grouped the sampling periods into three significantly different levels of abundance (Table 2), with larvae being most (and similarly) abundant in sampling periods that fell during January 1997, late June and July 1997, October–November 1997, and September–December 1998 (Figure 1). Larvae were least abundant in March to early May 1997, July 1997, January–May 1998, and July and August 1998 (Figure 1). When this grouping of sample periods was included, differences between years, seasons, and the periods observed by Randolph became nonsignificant l (chi square=4.13, df =3, p>0.2).

**Table 2:** Differences in abundance of larval and nymphal *Ixodes trianguliceps* ticks, counted on rodents of the study, in groups of sample periods deduced from the most parsimonious negative binomial regression models of the variations in tick abundance among sample periods^a^

Months	chi square	df	p value
**Larvae**			
Month group 2 vs. 1	5.21	1	<0.03
Month group 3 vs. 1	68.34	1	<0.001
Month group 3 vs. 2	6.25	1	<0.025
**Nymphs**			
Month group 2 vs. 1	53.75	1	<0.001

^a^Rodent ID was included in the models as a random effect. For larvae, month group 1 = March to early May 1997, July and December of 1997, January to late May, and July and August of 1998; month group 2 = February, August and September of 1997, and June of 1998; month group 3 = January, late May, June, October and November of 1997, and September to December 1998. For nymphs, month group 1 = January–April, September, November, and December of both 1997 and 1998; month group 2 = early May to August and October of both 1997 and 1998.

For nymphs, numbers counted were significantly higher in those sample periods that included months when nymphs were most abundant in the studies of Randolph (coefficient=1.907, SE=0.281, p<0.001), but again this finding only partly explained the between-sampling variation in the present study. In the most parsimonious model, significantly more nymphs were counted on the rodents in winter than in other months (coefficient=2.477, SE=1.027, p=0.016), and in the more detailed analysis, the most parsimonious model grouped the sampling periods into two significantly different levels of seasonal abundance (Table 2). We found no significant difference between years in the numbers of nymphs counted on the rodents (p>0.5) and when nymphs were most abundant in May–September and November in both years (Figure 1). Differences between seasons and the months of nymphal abundance observed by Randolph became nonsignificant when this grouping of sample periods was included in the same model (chi square=2.73, df=2, p>0.25).

Low numbers of adult female ticks were counted on the rodents. Although no significant differences were found among sample periods in their abundance, the raw data suggested that adult ticks were more abundant in early summer and autumn in both years than at other times (Figure 1).

Based on these findings, scales of a seasonal likelihood that a rodent encountered a larva or nymph (three- and two-point scales for larvae and nymphs, respectively) were included as explanatory variables in the second stage of the analysis. We found that heavier rodents carried greater numbers of ticks of any stage (coefficients=0.029, 0.099, and 0.170; p=0.033, 0.001, and 0.002, for larvae, nymphs, and adults, respectively; Table 3). Male bank voles carried significantly more larvae than did female bank voles and wood mice (coefficient=0.580, SE=0.279, p=0.037; Table 3). Wood mice of either sex carried significantly more larvae than female bank voles (coefficient=0.462; SE=0.229; p=0.044; Table 3). Male bank voles carried significantly more nymphs than did female bank voles or wood mice of either sex (coefficient=2.394; p=0.004; Table 3). Although some confounding between rodent mass and age category occurred, the latter was not significantly associated with variations in tick infestations (p>0.1 in all models). In models in which the scales of seasonal likelihood were excluded, almost all rodent-level factors remained significant, with the exception of the relationship between the counted numbers of larvae and rodent weight (p<0.334).

**Table 3:** Determinants of parasitism of rodents by larval, nymphal, and adult *Ixodes trianguliceps* ticks in the most parsimonious negative binomial regression models^a^

Variable	Coefficient	SE^b^	p value
**Larvae**			
Rodent body mass (g)	0.029	0.013	0.03
Month (3-point scale)	0.712	0.063	<0.001
Male bank voles vs. wood mice and female bank voles	0.580	0.279	0.04
Wood mice vs. female bank voles	0.462	0.229	0.04
Intercept	–0.702	0.468	
**Nymphs**			
Rodent body mass (g)	0.097	0.030	0.001
Month (2-point scale)	1.761	0.250	<0.001
Male bank voles vs. wood mice and female bank voles	2.394	0.832	0.004
Intercept	–6.590	0.915	
**Adults**			
Rodent body mass (g)	0.170	0.055	0.002
Intercept	–3.769	1.326	

^a^Rodent ID included as a random effect.

^b^SE, standard error.

Accounting for the seasonal likelihood of encountering a larva or nymph and rodent weight, sex, and species, a significant, positive relationship existed between the numbers of larvae and nymphs that fed on individual rodents (coefficient=0.373, SE=0.123, p=0.002). No significant relationships existed between the numbers of adult and larval ticks nor between the numbers of adult and nymphal ticks carried by the rodents (p>0.5 for both).

*A. phagocytophila* Infections in Rodents

Of 1,429 rodent blood samples tested, 527 were collected from bank voles and 902 from wood mice. Of these, 26 (5%) samples from bank voles (11%; 23/201 individual animals) and 7 (0.8%) samples from wood mice (1.8%; 7/390 individual animals) were PCR positive for *A. phagocytophila*. Analysis showed the sequence of bank vole origin (GenBank accession no. AY082656) was 99.9% similar to previously published sequences (e.g., GenBank accession no. AF470701.1); the sole difference was a guanine at base 33 in place of an adenine.

**Figure 2:**
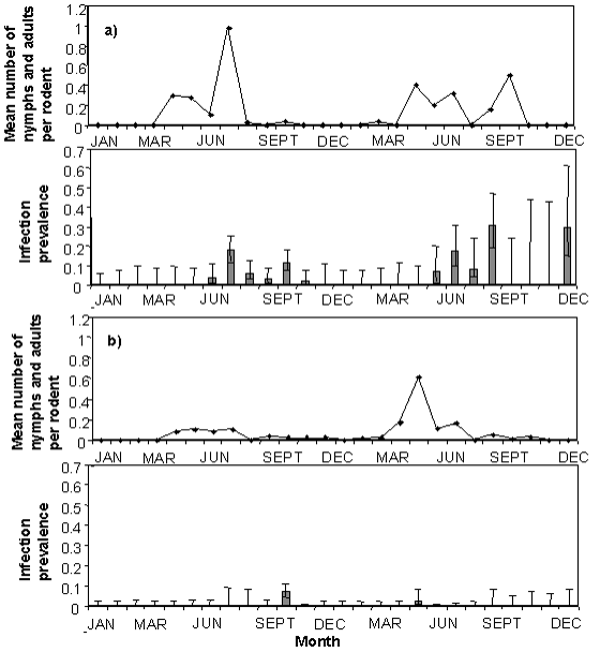
Prevalence of infection of *Anaplasma phagocytophila* (bar graphs +/- exact binomial errors) in blood samples collected from bank voles (graph marked a) and wood mice (graph marked b) compared to the mean monthly numbers of nymphal and adult *Ixodes trianguliceps* ticks counted per rodent at the time blood samples were collected (line graphs), 1997–1998.

Only blood from rodents captured during the periods June–November 1997, May–August 1998, and December 1998 was PCR positive (Figure 2). The highest prevalence of infection among bank voles was 30% (3/10 rodents in August 1998). The highest prevalence of infection among wood mice was 7.5% (6/80 rodents in October 1997). Blood from one bank vole was PCR positive in three consecutive sample periods, and blood from another was positive in two consecutive periods. One bank vole had PCR-positive blood on two occasions but had PCR-negative blood in the intervening sample period. Most rodents, however, had PCR-positive blood at only one sample period either because they were not trapped again after being positive (1 wood mouse and 9 bank voles) or because the results were negative at the subsequent sample period (6 wood mice and 14 bank voles). Of all rodents with PCR-positive blood, four bank voles and five wood mice had been captured and had negative results by PCR at more than one subsequent sampling period (a mean of four sample periods for the wood mice and five for the bank voles).

Univariate analyses showed bank voles were significantly more likely to have been PCR positive than wood mice (odds ratio [OR] 8.15, 95% confidence interval [CI] 3.08 to 21.59, p<0.001), and rodents were significantly more likely to be PCR positive if they carried a nymph (OR 5.49, 95% CI 1.62 to 18.54, p=0.006) or carried an adult tick (OR 9.25, 95% CI 2.10 to 40.84, p=0.003). Indices of the seasonal likelihood that rodents encountered a nymphal (as described above) or an adult tick (whether or not adult ticks were observed on any rodent in that sample period) were also significantly and positively associated with the likelihood that rodents were PCR positive (OR 4.9, CI 1.54 to15.86, p=0.007; OR 8.84, CI 2.74 to 28.47, p<0.001 for nymphs and adults, respectively). In the most parsimonious multivariable model, bank voles remained significantly more likely to be PCR positive and rodents were significantly more likely to be PCR positive if they carried a nymph or carried an adult (Table 4), although there was considerable confounding between the latter two factors and the indices of seasonal likelihood that rodents encountered nymphal or adult ticks. Seven (30%) of the 23 bank voles that had PCR-positive blood on the first occasion carried a nymphal or adult tick at time of sampling or at the previous sampling. In comparison, over the whole study, 80 (13%) of 598 captured bank voles and 162 (10%) of 1,698 of all rodents captured carried a nymph or and adult tick. Two PCR-positive bank voles carried a nymph or adult at the time of sampling only, three carried a nymph or adult at the previous sampling only, and two carried a nymph or adult at both samplings. None of the PCR-positive wood mice carried nymphs or adults at either sampling. No significant associations (p>0.1) were found between detected rodent infections and the presence of fleas at the time of sampling or if they also had a history of carrying a flea at the previous sampling. None of the other variables investigated, including interactions, was significantly associated with detected infection in the rodents in any of the models (p>0.1 for all). All significant factors remained so in models in which data from repeat-positive rodents were excluded.

**Table 4:** Relationships between individual variables and polymerase chain reaction result of rodent blood samples in the most parsimonious, minimal multivariable logistic regression model^a^

Variable	Coefficient (SE)	z	p value	Odds ratio	95% CI
Bank voles vs. wood mice	1.894 (0.468)	4.047	<0.001	6.65	2.66 to 16.64
Carried a nymphal tick	1.239 (0.556)	2.228	0.03	3.45	1.16 to 10.28
Carried an adult tick	2.369 (0.735)	3.224	0.001	10.69	2.53 to 45.09

^a^SE, standard error; CI, confidence interval.

*A. phagocytophila* Infection in *I. trianguliceps* Ticks

Of 59 *I. trianguliceps* ticks tested for *A. phagocytophila* infection, 39 were larvae, 7 were nymphs, and 13 were adult females. One (2.6%) of the larvae and 2 (15.3%) of the adult females tested positive, but none of the nymphs did. The PCR-positive larva was collected from one PCR-negative wood mouse captured in November 1997, whereas the positive adults came from one PCR-positive bank vole and one PCR-negative wood mouse captured in May 1998.

## Discussion

This study provides strong evidence that *A. phagocytophila* can be maintained in a system in which woodland rodents are a dominant reservoir host species and further suggests that *I. trianguliceps* is a competent vector. In addition, this study increases our understanding of the ecology of *A. phagocytophila* in a natural system. Detectable rodent infections were highly seasonal: PCR-positive rodents were detected from summer through autumn in both years of the study, but not from January to April in either year. This seasonality in infection prevalence appears to be associated with seasonal increases in the abundance of *I. trianguliceps* nymphs and adults, but not larvae. This finding is consistent with transstadial, but not transovarial, maintenance of *A. phagocytophila* by *I. trianguliceps* as appears to be the case for its other ixodid tick vectors (*3*,*6*,*12*). These findings, together with the detection of PCR-positive adult ticks, suggest that *I. trianguliceps* is a competent vector of *A. phagocytophila*.

Although individuals of both the common rodent species present in this woodland were PCR positive, bank voles were significantly more likely to be so (approximately eightfold) than wood mice, and positive wood mice were only detected in 1 month in each year. These differences may have been due in part to the greater numbers of nymphs carried by bank voles (approximately fourfold) than by wood mice. Differences in the roles of these two species as hosts for different developmental stages of *I. trianguliceps* have been recorded previously (*22*,*28*). In the present study, male bank voles carried a significantly greater proportion of nymphal and larval ticks, and heavier rodents of either species were more likely to carry a tick of any stage. These relationships imply that the lower resistance of reproductively active male bank voles for ticks (including *I. trianguliceps*; [*29*]) could be an explanatory factor, but other behavioral characteristics (e.g., resident rather than dispersing) (*30*) may have made them more likely to encounter ticks. Even when interspecific differences in contact rates with nymphal *I. trianguliceps* are allowed for, however, bank voles were significantly more likely to be detected as infected with *A. phagocytophila* than were wood mice. The course of infection in the two species may, therefore, be different. Although the majority of infections appeared to be transiently detectable, as are most infections in white-footed mice (*31*), 3 of the 22 PCR-positive bank voles were positive for more than one 4-week period, suggesting that *A. phagocytophila* infections may be more persistent in bank voles. If bank vole infections are more persistent, then by sampling every 4 weeks we may have missed proportionally more infections in wood mice than in bank voles.

The seasonal variations in the abundance of larval and nymphal *I. trianguliceps* in this study were very similar to those observed by Randolph (*21*), with some differences in detail. In both studies, larvae were most abundant from August to December or January, with a shorter period of activity in early summer that varied in amplitude between years. Nymphs were most abundant from May to August with some activity continuing through autumn. Although the numbers of adults were very low in this study, their seasonal appearance also corresponded to the findings in Randolph’s study. The similarities of the results of these and other studies (*22*–*24*) suggest that the observed variations in tick abundance may represent a more general seasonally repeated pattern of *I. trianguliceps* abundance, driven by the temperature-dependent tick development times deduced by Randolph (*21*). In this case, any cycles of *I. trianguliceps*–transmitted *A. phagocytophila* infection in the woodland may have comprised two components: a rapid within-year midsummer to early autumn component because of rapid intersstadial development of ticks influenced by higher summer temperatures (Figure 1A), and a longer component from autumn one year to spring/summer the next year because of lower intersstadial development rates influenced by low winter temperatures (Figure 1B).

The relatively short duration of *A. phagocytophila* infections in these rodents may have more general implications for the nature of endemic cycles involving rodents and the occurrence of rodent-derived infected ticks to which humans may be exposed in Europe. First, infected ticks are more likely to have been the most important overwinter reservoir of *A. phagocytophila* than the rodents, particularly as the tick development period over the winter may have been at the limit of the life expectancy of the rodents (*16*), a factor that may limit overwinter survival of rodent *Borrelia burgdorferi* sensu lato infections in some foci in northern Europe (*32*). This implication contrasts with the role of some rodent reservoirs such as the dusky-footed wood rat (*Neotoma fuscipes*) in the United States, which can remain PCR-positive for more than 1 year and act as an overwinter reservoir of infection (*33*,*34*).

Second, in experimentally infected rodents, efficient *A. phagocytophila* transmission to ticks occurs for only a short period because transmission is inhibited by the onset of acquired host resistance (*35*), a characteristic shared by tick-borne encephalitis virus (TBEV) (*36*) but not *B. burgdorferi* s.l. infections in rodents (*37*). Because of such short periods of infectivity, the occurrence of endemic cycles of TBEV depends on coincident seasonal activities of different *I. ricinus* tick instars, coupled with aggregation of ticks of more than one instar on a small proportion of the rodent population (*38*). In our study, seasonal activities of larvae, nymphs, and adults were partly coincident, the distribution of ticks among rodents was highly aggregated, and larvae and nymphs co-fed on a small proportion of the population (particularly bank voles), conditions that may have enhanced transmission of short-lived *A. phagocytophila* infections. Such conditions may also promote co-feeding transmission of *A. phagocytophila* (*35*): the detection of infection in a larval tick collected from a PCR-negative rodent may suggest that this transmission route occurs naturally on rodents. European rodents may, therefore, be important reservoirs of *A. phagocytophila*, but the risk of rodent-derived human infections may be constrained by factors that also constrain the risk of TBEV infection when endemic cycles are maintained by exophilic *I. ricinus* ticks alone.

In this study, cycles of infection were maintained even though the mean numbers of *I. trianguliceps* per rodent were very low (never >1 for any instar). Therefore, when *I. trianguliceps* and *I. ricinus* ticks are sympatric, *I. trianguliceps*–driven endemic cycles may provide an efficient reservoir from which *I. ricinus* may acquire infections from rodents, thus increasing the risk of rodent-derived human infections. In this respect, rodent-*trianguliceps* cycles may have a similar role in *A. phagocytophila* maintenance in Europe to that of cycles maintained in dusky wood rats and nidicolous *I. spinipalpis* ticks in the western United States where sympatric exophilic *I. pacificus* ticks are the bridge vector transmitting infections to humans and domesticated animals (*33*). This maintenance system may be particularly important in Great Britain, where woodland rodents carry few nymphal or adult *I. ricinus* (*39*) and, in the absence of *I. trianguliceps*, rodent *A. phagocytophila* infections may be uncommon (*11*). Further studies are required to test these hypotheses and investigate the role of rodent-derived *A. phagocytophila* in human infections in Europe.
